# Timing matters: a life-course review of midlife type 2 diabetes, exposure duration, and late-life dementia risk

**DOI:** 10.1007/s40200-026-02111-2

**Published:** 2026-10-17

**Authors:** Mrityunjay Sivakumar, Chakravarthy Marx Sadacharan

**Affiliations:** 1 College of Sciences, Georgia Institute of Technology, 225 North Ave NW #202, Atlanta, GA 30332 USA; 2 Department of Biomedical Sciences, Anatomy & Embryology, Tilman J. Fertitta Family College of Medicine (TJFFCOM), University of Houston, 5055 Medical Cr, Houston, TX 77204 USA

**Keywords:** Type 2 diabetes mellitus, Dementia, Midlife diabetes, Hypoglycemia, Alzheimer’s disease, Vascular dementia

## Abstract

**Purpose:**

This review synthesizes the evidence regarding Type 2 diabetes mellitus (T2DM) as a critical modifiable risk factor for dementia through a life-course lens. The primary purpose is to move beyond the general association and determine how the timing and severity of T2DM, specifically age at onset, duration of disease, and glycemic instability, influence cognitive decline. Consequently, this paper seeks to answer the central research question: How do midlife versus late-life T2DM onset and cumulative glycemic exposure independently contribute to the long-term risk and biological pathways of late-life dementia?

**Methods:**

A structured narrative review was conducted using predefined search strategies and study selection criteria, focusing on peer-reviewed studies published 2020–2025 and key longitudinal cohorts. Studies included large prospective cohorts, population-based analyses, or meta-analyses reporting hazard ratios (HRs), relative risks (RRs), or odds ratios (ORs) for incident dementia, Alzheimer’s disease (AD), or vascular dementia (VaD). Key exposures included midlife onset, duration, cumulative glycemic measures, and severe hypoglycemia.

**Results:**

T2DM was associated with a 1.3–1.8-fold increased risk of incident dementia. Earlier onset and longer duration showed graded risk increases, while higher cumulative glycemic burden and severe hypoglycemia were linked to greater dementia incidence. Associations were stronger for VaD than AD.

**Conclusions:**

Midlife T2DM appears to be a strong exposure-dependent risk factor for late-life dementia. Preventing T2DM onset, maintaining stable glycemic control, and minimizing hypoglycemia may be associated with lower future dementia risk, pending confirmation in interventional studies.

## Introduction

Dementia has emerged as one of the most pressing public health challenges of the 21st century. As global populations continue to age, the prevalence of dementia is expected to rise substantially, placing increasing pressure on health care systems, long-term care infrastructure, and socioeconomic stability. The 2024 report from the Lancet Standing Commission on Dementia Prevention, Intervention, and Care highlights that dementia is now a leading cause of disability and dependency worldwide and emphasizes the importance of identifying modifiable risk factors across the life course [1]. As a result, dementia is increasingly viewed not solely as a condition of late life but as the culmination of biological and environmental exposures that begin decades earlier.

Among these modifiable risk factors, type 2 diabetes mellitus (T2DM) has gained significant attention. Over the past several decades, the global prevalence of T2DM has risen dramatically, to an estimated 828 million adults in 2022, an increase of approximately 630 million since 1990, largely driven by aging populations, increasing rates of obesity, sedentary lifestyles, and the growing burden of metabolic syndrome [2]. At the same time, epidemiologic evidence has consistently shown that individuals with T2DM have a higher risk of cognitive decline and dementia [3–5]. Large-scale meta-analyses demonstrate elevated risk of all-cause dementia, Alzheimer’s disease (AD), and vascular dementia (VaD) among individuals with T2DM, with pooled relative risks of approximately 1.3–1.8 [3, 4]. In a recent nationwide Korean cohort of more than 1.3 million adults, adjusted hazard ratios (HRs) ranged from 1.29 among individuals with T2DM treated with oral glucose-lowering agents to 2.14 among those treated with insulin for all-cause dementia, from 1.29 to 2.18 for AD, and from 1.29 to 2.23 for VaD [6]. Importantly, this risk appears to be shaped not only by the presence of T2DM but also by glycemic dynamics, including chronic hyperglycemia, glycemic variability, and hypoglycemia [7–11].

More recently, research in this area has moved beyond simply establishing that T2DM increases dementia risk. A more nuanced perspective has emerged, suggesting that T2DM is not a uniform exposure and that its cognitive consequences vary depending on factors such as age at onset, duration of disease, metabolic severity, and treatment context, that is, whether glycemia is managed with lifestyle measures alone, oral glucose-lowering agents, or insulin, and whether the regimen carries a risk of hypoglycemia (e.g., insulin or sulfonylureas). Insulin-treated T2DM, which generally reflects more advanced or longer-standing disease, has been associated with roughly twice the dementia risk of T2DM managed with oral agents [6]. The life-course epidemiology framework provides an important lens for interpreting these findings. Rather than viewing T2DM as a binary condition measured at a single time point, it is increasingly recognized as a dynamic exposure that develops over time and interacts with vascular, metabolic, inflammatory, and neurodegenerative processes.

Within this framework, midlife appears to represent a particularly important window of vulnerability. Longitudinal cohort studies have shown that vascular risk factors present during midlife, including T2DM, are associated with the development of dementia decades later. Large prospective cohort studies such as the Atherosclerosis Risk in Communities (ARIC) study demonstrated that midlife T2DM and related vascular risk factors predict incident dementia in later life [12]. More recent analyses have expanded on this by examining gradients in age at T2DM onset, with findings suggesting that prolonged exposure to metabolic dysregulation, rather than T2DM status at a single point in time, plays a key role in shaping long-term neurocognitive risk.

Evidence related to T2DM duration further supports this life-course model. Several cohort studies have shown that longer duration of T2DM is associated with progressively higher risk of dementia. For instance, Reinke et al., using German health claims data, found that dementia incidence increased with longer duration of T2DM [13]. Updated meta-analytic findings from Cao et al. indicate that even relatively short durations of diabetes (≤ 5 years) are associated with elevated dementia risk, with stronger associations observed as exposure length increases [3]. These findings are consistent with a cumulative exposure model, in which chronic hyperglycemia, insulin resistance, and vascular injury exert additive effects on the brain over time.

In addition to timing and duration, glycemic dynamics have also emerged as important modifiers of dementia risk. Long-term glycemic burden appears to be more relevant than single measurements of glucose control. In a large longitudinal cohort, Moran et al. demonstrated that cumulative glycemic exposure across several decades was strongly associated with dementia incidence among individuals with T2DM [7]. Similarly, Underwood et al. reported that greater time spent within a glycated hemoglobin (HbA1c) target range was linked to lower dementia risk among older adults with diabetes [8]. Glycemic variability has also been implicated: in a U.K. primary-care cohort of more than 450,000 adults with diabetes, greater visit-to-visit variability in HbA1c was associated with higher subsequent dementia risk [11]. These findings suggest that sustained metabolic stability and low glycemic variability may play a protective role and highlight the importance of evaluating chronic glycemic exposure rather than isolated clinical measures.

Another factor receiving increasing attention is hypoglycemia, which has been identified as an independent and potentially bidirectional contributor to cognitive decline. Severe hypoglycemic events have been associated with incident dementia in large population-based cohorts [9]. A systematic review published in 2025 further supported hypoglycemia as a risk factor for dementia among individuals with diabetes [10]. Mechanistically, hypoglycemia may lead to neuronal energy failure, oxidative stress, and microvascular injury, thereby compounding the effects of chronic hyperglycemia. These findings complicate the traditional view that tighter glycemic control is always beneficial and instead emphasize the importance of individualized treatment targets, particularly among older adults [14].

Mechanistic research provides biological plausibility for these epidemiologic associations. Insulin resistance, oxidative stress, inflammation, blood-brain barrier dysfunction, and microvascular injury have all been implicated as potential pathways linking T2DM to neurodegeneration. These mechanisms are discussed in greater detail below [15–19].

More recent pharmacologic studies have introduced an additional dimension to this relationship. Observational data indicate that certain glucose-lowering therapies may influence dementia risk. For example, a large population-based cohort study found that initiation of sodium–glucose cotransporter-2 (SGLT2) inhibitors was associated with lower dementia risk compared with dipeptidyl peptidase-4 (DPP-4) inhibitors [20]. Additionally, systematic reviews and meta-analyses published in 2025 suggest that SGLT2 inhibitors and glucagon-like peptide-1 receptor agonists (GLP-1 RAs) may be associated with reduced risk of cognitive decline and dementia [21, 22]. Although these findings are primarily observational, they raise important questions about whether targeting metabolic and inflammatory pathways could alter the trajectory of neurodegenerative disease.

While prior reviews have examined the general association between T2DM and dementia, few have systematically synthesized age-at-onset gradients, duration-dependent risk, hypoglycemia, and pharmacologic modifiers within a unified life-course framework specific to T2DM. By contrast, in type 1 diabetes, more than three decades of follow-up in the Diabetes Control and Complications Trial and its observational extension, the Epidemiology of Diabetes Interventions and Complications study (DCCT/EDIC), have already characterized how cumulative glycemic exposure and severe hypoglycemia relate to cognitive decline [23]. For T2DM, several important gaps remain. Earlier reviews often did not clearly distinguish between midlife and late-life T2DM, did not quantify age-at-onset gradients, and did not fully integrate newer evidence related to duration-specific risk, hypoglycemia, and pharmacologic modifiers. In addition, recent research highlights notable racial and ethnic disparities in the relationship between diabetes and dementia [24, 25], yet these disparities have not been fully incorporated into broader life-course models.

Accordingly, this review synthesizes recent epidemiologic, mechanistic, and pharmacologic evidence published between 2020 and 2025, while also incorporating key foundational longitudinal studies. Specifically, it advances three central arguments: (1) age at T2DM onset is a major determinant of lifetime dementia risk; (2) longer duration and greater cumulative glycemic burden contribute to graded and biologically plausible increases in risk; and (3) hypoglycemia represents an emerging and clinically actionable risk amplifier. By integrating mechanistic insights with refined epidemiologic patterns, this review situates midlife T2DM within a broader life-course framework of neurodegeneration and highlights important opportunities for prevention, clinical management, and future research.

## Methods

### Study design and conceptual framework

This study was conducted as a structured narrative review with structured narrative synthesis to synthesize contemporary evidence on the association between T2DM and incident dementia. The review was guided by a life-course epidemiology framework that focused on three primary exposure dimensions: (1) age at T2DM onset, (2) duration of T2DM and cumulative glycemic burden, and (3) hypoglycemia and glycemic variability as potential modifiers of risk. These three dimensions are not independent risk factors but interlocking components of a single cumulative exposure process: age at onset determines the potential duration of exposure, and duration in turn determines the time window over which glycemic burden can accumulate. Cumulative glycemic exposure can therefore be understood as the integrated ‘dose’ of metabolic dysregulation, analogous to pack-years in smoking research, with hypoglycemia acting as an additional, partially independent modifier of risk. Mechanistic and pharmacologic studies were also included to provide biological and clinical context for observed epidemiologic patterns.

### Literature identification and selection

Searches were conducted between January 1 and February 28, 2026. Peer-reviewed articles published between January 2020 and March 2025 were prioritized to incorporate recent epidemiologic, mechanistic, and pharmacoepidemiologic evidence. Foundational studies published prior to 2020 were included when necessary to contextualize current findings.

Studies were identified through structured searches of PubMed and Google Scholar, supplemented by citation tracking of relevant reviews, meta-analyses, and major cohort studies. Search terms included combinations of “type 2 diabetes,” “midlife diabetes,” “age at onset,” “diabetes duration,” “glycemic control,” “hypoglycemia,” “cognitive decline,” “Alzheimer’s disease,” “vascular dementia,” and “incident dementia.”

Inclusion criteria prioritized prospective cohort studies with longitudinal follow-up, population-based registry analyses, large administrative database studies, systematic reviews and meta-analyses, and pharmacoepidemiologic investigations evaluating dementia risk by medication class.

Titles and abstracts were screened for relevance based on predefined inclusion criteria. Full-text review was performed for studies meeting screening criteria. The search identified approximately 120 records; after title/abstract screening, 51 were selected for full-text review, of which 14 met inclusion criteria and were incorporated into the narrative synthesis. Given the narrative design of the review, formal duplicate independent screening was not conducted.

Studies were required to report quantitative effect estimates, including hazard ratios (HRs), relative risks (RRs), or odds ratios (ORs), with corresponding 95% confidence intervals. Studies focused solely on type 1 diabetes were excluded. Studies including participants with unspecified diabetes type were retained if they otherwise met inclusion criteria.

### Exposure definitions

T2DM exposure was defined according to each study’s criteria, including physician diagnosis, medication use, fasting glucose thresholds, HbA1c, or administrative diagnostic coding. Midlife T2DM was generally defined as onset between ages 40 and 60 years, although definitions varied slightly across studies. When available, analyses stratified by age at diagnosis were extracted.

Duration of T2DM was assessed as reported in individual studies and was commonly categorized as ≤ 5 years, 6–10 years, and > 10 years, or modeled as a continuous variable. Measures of cumulative glycemic exposure, including long-term HbA1c averages or time-in-range metrics, were extracted when reported.

Hypoglycemia was defined according to study-specific criteria, most frequently as severe events requiring medical attention or hospitalization.

### Outcome definitions

The primary outcome was incident all-cause dementia. Secondary outcomes included Alzheimer’s disease and vascular dementia when subtype-specific diagnoses were reported. Dementia ascertainment methods varied across studies and included clinical adjudication, neurocognitive testing, medical record review, and administrative diagnostic coding.

### Data extraction and synthesis

For each included study, the primary reported effect estimate was extracted. For overall associations between T2DM and dementia, minimally adjusted models were reported when they reflected total risk burden. For analyses examining age at onset, duration of T2DM, glycemic control, or hypoglycemia, fully adjusted multivariable models were prioritized to account for demographic and vascular confounders.

Because of heterogeneity in exposure definitions, dementia ascertainment methods, and analytic approaches across studies, formal quantitative pooling was not performed. Given the substantial heterogeneity in exposure definitions (age cutoffs, duration categorization), outcome ascertainment (clinical adjudication versus administrative coding), and adjustment strategies, pooled quantitative estimates were considered potentially misleading and were therefore not undertaken. Instead, findings were synthesized descriptively with attention to consistency of effect estimates and reported associations across studies.

Mechanistic and translational studies were integrated narratively to provide biological context for epidemiologic findings.

### Generalizability and population diversity

To enhance external validity, studies were included from diverse geographic regions, including North America, Europe, and Asia, as well as multinational pooled analyses. Where available, subgroup analyses by race, ethnicity, and sex were incorporated to examine heterogeneity in risk patterns. Where available, sex-stratified analyses were reviewed; however, sex-specific findings were inconsistently reported across studies.

### Limitations of the review approach

This review did not involve new data collection or statistical re-analysis. Findings reflect reported effect estimates from included studies and may be influenced by heterogeneity in study design, exposure measurement, and outcome ascertainment. The absence of formal quantitative pooling was intended to avoid aggregation across methodologically diverse cohorts.

A formal PRISMA flow diagram and quantitative risk-of-bias assessment were not undertaken, as the objective was conceptual synthesis rather than systematic meta-analysis.

## Results

### Association between midlife type 2 diabetes and late-life dementia risk

Across prospective cohort studies and contemporary meta-analyses, midlife T2DM is consistently associated with elevated risk of incident late-life dementia. Updated pooled estimates confirm a robust association across all-cause dementia, Alzheimer’s disease (AD), and vascular dementia (VaD).

In a 2024 meta-analysis synthesizing prospective cohorts, Cao et al. reported elevated dementia risk across multiple analytic models, with pooled relative risks generally ranging between approximately 1.3 and 1.8 depending on adjustment strategy and population with elevated risks also observed for AD and VaD [3]. Other meta-analyses demonstrate similar magnitudes, reinforcing temporal consistency of effect estimates [4]. Across syntheses, the association is typically stronger for vascular dementia than for AD.

**Fig. 1:**
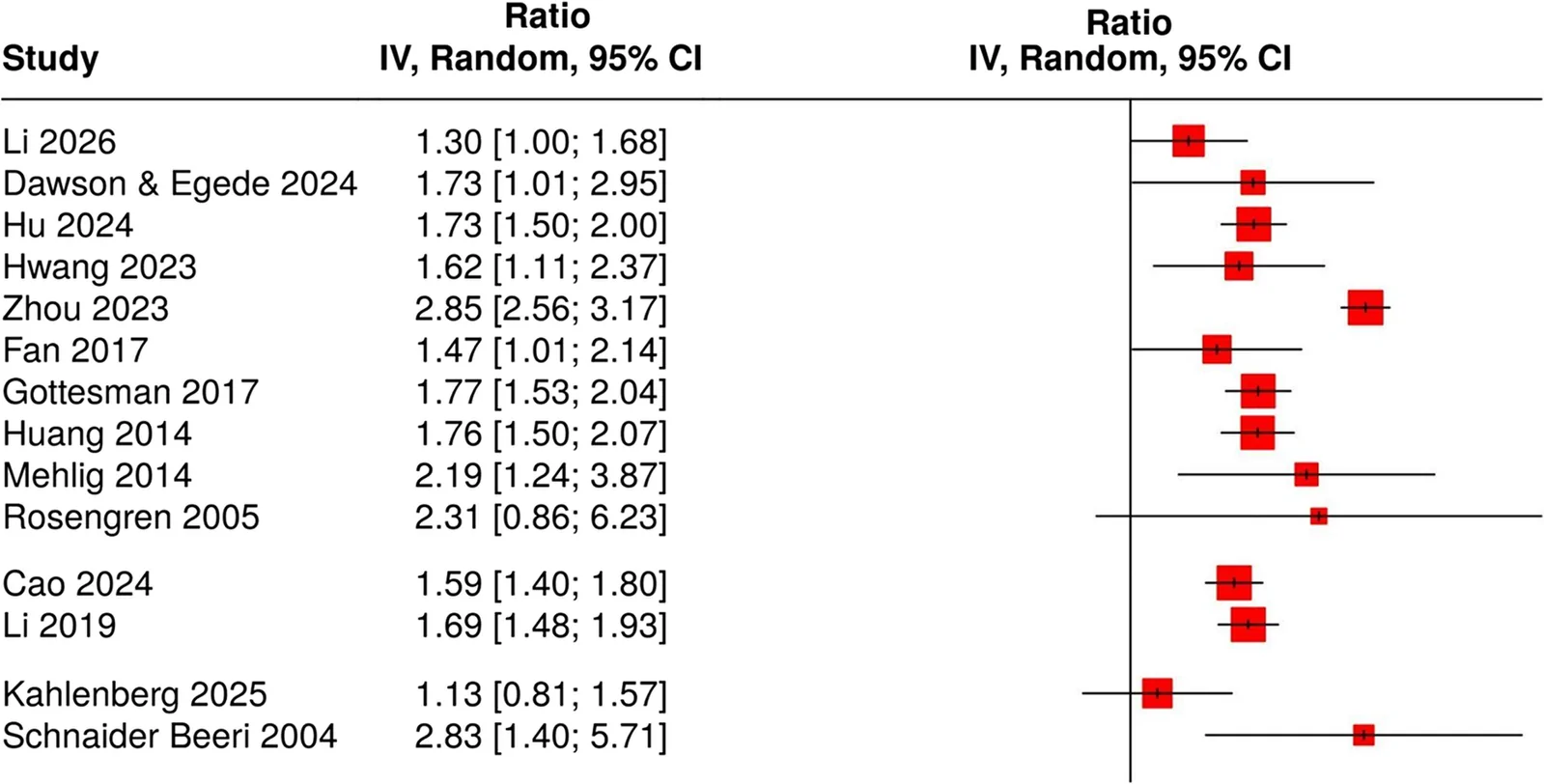
Forest plot of midlife type 2 diabetes and incident late-life dementia. Forest plot displaying hazard ratios (HRs), relative risks (RRs), and odds ratios (ORs) with 95% confidence intervals from selected prospective cohort studies and meta-analyses evaluating the association between midlife type 2 diabetes and incident all-cause dementia, separated in order of what their results used. Estimates reflect study-specific exposure definitions and adjustment models. This figure reproduces published hazard ratios and does not represent newly pooled estimates [12, 31–39]

Findings from large longitudinal cohort studies were consistent with these pooled analyses. In the ARIC study, midlife T2DM was associated with increased 25-year risk of incident dementia after multivariable adjustment [12]. Associations were observed across sex and race strata, although effect estimates varied across subgroups. A forest plot has been created that summarizes representative hazard ratios (HRs) and relative risks (RRs) from selected prospective cohorts and pooled analyses examining midlife T2DM and incident dementia (Fig. 1).

### Age at diabetes onset and lifetime dementia risk

Several cohort studies reported associations between age at T2DM onset and subsequent dementia incidence. In the Whitehall II cohort, Barbiellini Amidei et al. reported that, at age 70, each 5-year earlier T2DM onset was associated with a 24% higher hazard of dementia (HR 1.24; 95% CI 1.06–1.46) [26]. Compared with individuals without T2DM, those with T2DM duration greater than 10 years at age 70 had more than double the risk of dementia (HR 2.12; 95% CI 1.50-3.00) (Table 1).

**Table 1:** Age at onset and duration of diabetes in relation to dementia risk

Hazard ratio (95% CI)	No diabetes	Diabetes onset ≤ 5 years earlier	Diabetes onset 5–10 years earlier	Diabetes onset > 10 years earlier
55yo	1.00 (Ref)	2.14 (1.44–3.17)	—	—
60yo	1.00 (Ref)	1.77 (1.13–2.77)	2.19 (1.48–3.26)	—
65yo	1.00 (Ref)	1.53 (1.03–2.29)	2.03 (1.48–2.79)	—
70yo	1.00 (Ref)	1.11 (0.70–1.76)	1.49 (0.96–2.32)	2.12 (1.50-3.00)

Table adapted from Barbiellini Amidei et al. [26], presenting hazard ratios (HRs) with 95% confidence intervals for incident dementia according to age (55, 60, 65, and 70 years) and duration of diabetes (≤ 5 years, 6–10 years, > 10 years). Estimates are derived from multivariable-adjusted models and demonstrate graded increases in dementia risk with earlier onset and longer duration

In ARIC, Hu et al. demonstrated that earlier age at T2DM diagnosis was associated with substantially higher lifetime risk of dementia [27]. Participants diagnosed before age 60 exhibited higher cumulative dementia incidence compared with those diagnosed later, even after accounting for competing mortality.

Across cohorts, T2DM onset during midlife (generally defined as ages 40–60 years) was associated with higher subsequent dementia risk compared with late-life onset. These findings were consistent across analytic approaches using categorical and continuous age-at-onset models.

### Duration-specific risk and cumulative exposure

The duration of T2DM is an independent factor associated with dementia risk across several populations. In a German health claims cohort, Reinke et al. reported that dementia incidence increased as the duration of T2DM became longer [13]. Risk estimates rose progressively across duration categories, suggesting a clear duration–response relationship.

Similarly, pooled analyses by Cao et al. showed that dementia risk was already elevated within five years of diabetes diagnosis (RR 1.29; 95% CI 1.20–1.39), with stronger associations observed as duration increased [3]. Long-term glycemic exposure further clarifies this pattern. Moran et al., who examined cumulative glycemic control over multiple decades, found that higher long-term glycemic burden was associated with greater dementia incidence among individuals with T2DM [7]. These associations remained significant after adjusting for demographic and vascular covariates.

Overall, these findings indicate that dementia risk is not only linked to the presence of T2DM but also increases with longer exposure to metabolic dysregulation.

### Glycemic control, variability, and metabolic severity

Measures of metabolic severity also appear to influence dementia risk. Moran et al. showed that long-term glycemic control trajectories were associated with incident dementia, with poorer control corresponding to higher risk [7].

In another study of older adults with T2DM, Underwood et al. reported that greater HbA1c time-in-range was linked to a lower risk of incident dementia [8]. Individuals who spent more time within individualized glycemic targets experienced reduced dementia incidence compared with those who had greater exposure outside of target ranges.

Together, these findings highlight the importance of cumulative glycemic burden and stability in glycemic control as measurable factors that may modify cognitive risk.

### Hypoglycemia and dementia risk

Severe hypoglycemia has also been identified as an independent predictor of dementia. In a population-based cohort study, Alkabbani et al. found that severe hypoglycemic episodes occurring in both midlife and late life were associated with incident dementia [9]. The risk increased as hypoglycemic events occurred.

A 2025 systematic review by Konda et al. further confirmed the association between hypoglycemia and dementia risk among individuals with diabetes [10]. Across multiple studies, severe hypoglycemia was consistently linked with higher dementia incidence independent of baseline glycemic control.

In most cohorts, hypoglycemia exposure was defined as episodes requiring medical attention or hospitalization, meaning that milder or unreported events were not consistently captured.

### Dementia subtype-specific associations

When dementia was analyzed by subtype, T2DM was associated with increased risk for both AD and VaD. Meta-analytic evidence suggests that the association is generally stronger for vascular dementia compared with AD [3]. Although the relationship with AD remained consistently elevated, the magnitude of effect was smaller relative to VaD.

Subtype-specific analyses across cohorts showed variability in effect sizes, which may be influenced by differences in diagnostic criteria and follow-up duration. Several pooled analyses also reported outcomes involving mixed dementia.

### Pharmacologic modifiers of dementia risk

Recent pharmacoepidemiologic studies suggest that dementia risk may differ across glucose-lowering medication classes.

In a population-based cohort of adults aged 40–69 years with T2DM, Shin et al. found a lower risk of dementia among individuals initiating SGLT2 inhibitors compared with those initiating DPP-4 inhibitors [20]. In addition, systematic reviews and meta-analyses conducted in 2025 suggest that SGLT2 inhibitors and GLP-1 receptor agonists may be associated with reduced risk of dementia or cognitive decline compared with other therapies [21, 22].

These findings are based on observational studies using adjusted hazard ratios, and there is some heterogeneity across study populations and analytic approaches. They are also subject to confounding by indication, as the decision to initiate SGLT2 inhibitors or GLP-1 receptor agonists over other agents may itself reflect underlying differences in cardiovascular risk, renal function, weight status, or overall health that independently influence dementia risk.

### Mechanistic integration with epidemiologic findings

Translational studies provide biologically plausible mechanisms linking T2DM to dementia, including insulin resistance, oxidative stress, inflammation, and microvascular injury. These pathways are described in greater detail in the Discussion.

Raza et al. describe hyperglycemia-induced oxidative stress and breakdown of the blood–brain barrier as important contributors to AD pathology in T2DM [17]. Wang et al. (2020) report associations between T2DM and increased white matter hyperintensity burden, while Ly et al. demonstrate diabetes-related microvascular injury linked to white matter disease [16, 18].

**Fig. 2:**
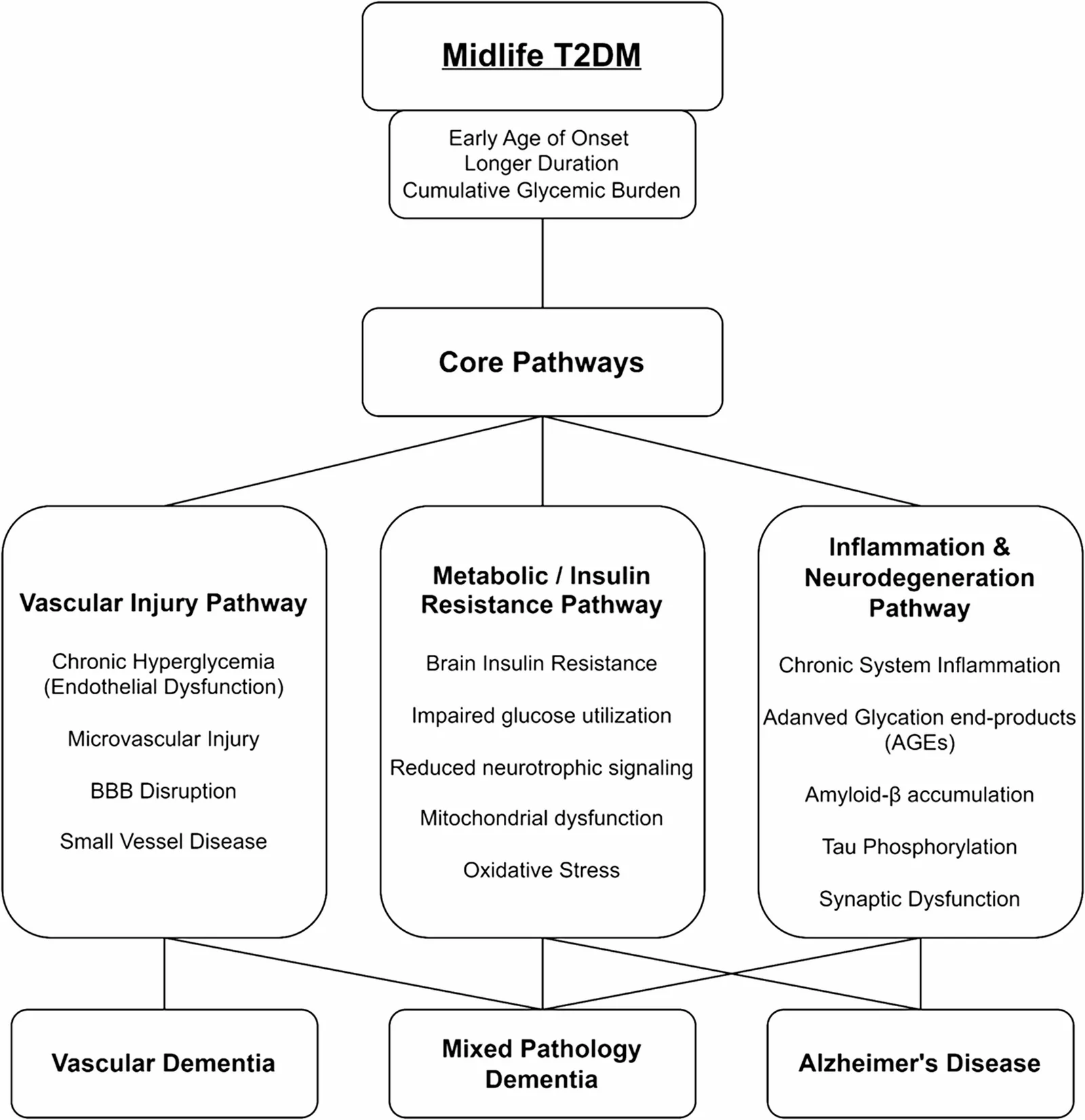
Mechanistic model linking midlife type 2 diabetes to late-life dementia. Conceptual mechanistic model illustrating three primary pathways linking midlife type 2 diabetes to late-life dementia: (1) vascular injury pathway (microvascular dysfunction, white matter hyperintensities, endothelial damage); (2) metabolic/insulin resistance pathway (impaired brain insulin signaling, amyloid-beta accumulation, tau phosphorylation); and (3) inflammation and neurodegeneration pathway (oxidative stress, cytokine activation, blood–brain barrier dysfunction). These mechanisms converge to increase risk of vascular dementia, Alzheimer’s disease, and mixed-pathology dementia

These mechanisms are summarized in Fig. 2.

### Methodological heterogeneity across studies

Considerable heterogeneity was observed across studies in definitions of midlife exposure (age cutoffs ranging approximately 40–65 years), diabetes ascertainment (self-report, medical records, fasting glucose, HbA1c, medication use), duration measurement (categorical vs. continuous), dementia ascertainment (clinical adjudication vs. administrative coding), covariate adjustment strategies, and competing risk modeling.

These differences introduce potential sources of bias, including exposure misclassification, variability in dementia subtype diagnosis, residual confounding, and differences in competing risk modeling. Such heterogeneity limits direct comparison of effect sizes and underscores the need for cautious interpretation of pooled estimates. Summaries of this information are located in Table 2.

**Table 2:** Methodological characteristics of studies included in forest plot

Article	Population	Relevant information for hazard ratio (HR) variance
Li (2026) [31]	Americans (*N* = 37,391)	Timing: Midlife onset poses higher relative risk than late-life. PAF: Highlights diabetes as a top modifiable risk factor.
Hwang (2023) [32]	Americans (MA) (*N* = 5,015)	Age-Dependency: Risk decreases when diagnosed in later life. Cohort age: Explains lower HRs in older study populations.
Zhou (2023) [33]	United Kingdom (*N* = 447,931)	Statistical Power: Massive sample size minimizes error. Cohort Profile: Robust “pure” comparison in Western population.
Li (2020) [34]	Global Meta-Analysis (*N* = 2.3 Million)	Aggregation: Weighted average of 144 global studies. Conservative Estimate: Smoothes out regional/cohort spikes.
Fan (2017) [35]	Taiwan (*N* = 5,161)	Independence: Risk remains after adjusting for hypertension. Data Type: Claims-based data may underestimate clinical severity.
Gottesman (2017) [12]	Americans (Black/White) (*N* = 15,792)	Race: Variations in risk across Black/White participants. Diabetes identified as the strongest midlife risk factor.
Huang (2014) [36]	Taiwan (*N* = 142,744)	Regionality: Baseline risk consistency in East Asian cohorts. Geography: Lower HR compared to Western/UK estimates.
Mehlig (2014) [37]	Sweden (Female) (*N* = 1,359)	Gender: Female-only cohort shows heightened vulnerability. Specificity: Gender differences in metabolic-driven cognitive decline.
Rosengren (2005) [38]	Sweden (Male) (*N* = 7404)	Sample Size: High HR but not statistically significant. CI Width: Small sub-group size leads to wide confidence intervals.
Schnaider Beeri (2004) [39]	Israeli (Jewish) (*N* = 1,892)	Ethnicity: Specific ethnic/genetic risk profiles. Monitoring: High HR likely due to close clinical monitoring

Table summarizing selected studies included in Fig. 1. Columns include: (1) Article, (2) Population and sample size, and (3) Relevant factors contributing to *HR* hazard ratio variance, *PAF*  Population Attributable Fraction. This table illustrates methodological heterogeneity across cohorts

## Discussion

### Principal findings in context

This review brings together recent epidemiologic, translational, and pharmacoepidemiologic evidence showing that midlife T2DM is consistently associated with an increased risk of late-life dementia. Across prospective cohort studies and updated meta-analyses, individuals with T2DM have consistently elevated risk of developing all-cause dementia across multiple cohorts. These associations are observed for both Alzheimer’s disease (AD) and vascular dementia (VaD), although the effect tends to be stronger for VaD [3, 12]. Overall, these findings align with earlier landmark studies and are reinforced by newer large-scale analyses.

Importantly, the findings summarized in the Results extend beyond simply identifying T2DM as a risk factor. Instead, several patterns emerge that help refine how this risk develops over time. In particular, earlier age at T2DM onset, longer duration of the disease, and metabolic instability, including hypoglycemia, appear to influence dementia risk. Together, these observations support a life-course perspective, where prolonged metabolic dysfunction beginning in midlife appears to be associated with increased neurodegenerative and cerebrovascular vulnerability later in life.

### Age at onset: a life-course gradient

Age at T2DM diagnosis appears to be an important factor in determining long-term cognitive risk. Evidence from the Whitehall II study showed that each 5-year earlier onset of T2DM was associated with a 24% higher risk of dementia by age 70 [26]. Similarly, analyses from the ARIC cohort indicate that individuals diagnosed before age 60 experience greater cumulative dementia incidence compared with those diagnosed later, even after accounting for competing mortality [27].

These findings suggest that T2DM is not simply a condition that increases dementia risk at any point in life. Instead, earlier onset may lead to a longer period of metabolic dysregulation that overlaps with decades of preclinical neurodegenerative changes. The consistency of this trend across multiple cohorts strengthens the idea that earlier exposure to T2DM may have a disproportionate impact on long-term cognitive outcomes.

### Duration-specific risk and cumulative glycemic burden

In addition to age at onset, the duration of T2DM also appears to play a significant role in dementia risk. Across several cohorts, longer duration of T2DM has been associated with progressively higher dementia incidence [3, 13]. For example, individuals who had lived with T2DM for more than 10 years by age 70 had more than double the risk of dementia compared with individuals without T2DM [26].

Longitudinal studies examining glycemic trajectories further support this cumulative exposure model. Greater long-term glycemic burden and poorer overall control have been linked with higher rates of incident dementia [7]. On the other hand, spending more time within target HbA1c ranges has been associated with lower dementia risk [8]. Taken together, these findings suggest that long-term metabolic patterns may be more informative than a single measurement of glycemic control when evaluating cognitive risk.

These findings support a model in which age at onset, duration, and cumulative glycemic burden operate as a single interconnected exposure rather than three separate risk factors. Earlier onset extends the potential duration of metabolic dysregulation, and that duration, combined with glycemic control quality, determines the total cumulative burden the brain is exposed to. This integrated framework helps explain why studies examining T2DM status alone tend to show weaker or less consistent associations than those accounting for timing and cumulative exposure together.

### Hypoglycemia as an independent and amplifying risk factor

Beyond chronic hyperglycemia, hypoglycemia has emerged as another important factor associated with dementia risk. Severe hypoglycemic events were independently associated with incident dementia in population-based cohorts [9], and systematic reviews have confirmed this relationship across multiple study designs [10].

Some studies also suggest a dose-response relationship, where more frequent severe hypoglycemic episodes correspond to higher dementia incidence. However, most analyses only capture episodes requiring medical attention, which means the total burden of hypoglycemia may be underestimated.

These findings highlight a potentially complex aspect of T2DM management. While chronic hyperglycemia is associated with long-term metabolic injury, overly aggressive glucose lowering, especially in older adults, may increase vulnerability through acute neuronal energy deprivation and related stress responses. This dual risk underscores the importance of individualized treatment strategies.

It is also important to consider reverse causation: undiagnosed or early-stage cognitive impairment may itself increase the risk of severe hypoglycemia (for example, through impaired medication self-management, missed meals, or errors in insulin dosing) complicating causal interpretation of this association.

### Dementia subtypes: vascular and neurodegenerative convergence

Subtype analyses show that diabetes is associated with increased risk of both AD and VaD, although the association is generally stronger for VaD [3]. This pattern is consistent with well-established effects of T2DM on vascular health, including microvascular damage and endothelial dysfunction.

At the same time, the consistent relationship between T2DM and AD suggests that metabolic factors may influence neurodegenerative processes beyond vascular mechanisms alone. Many studies also report mixed dementia outcomes, supporting the idea that multiple pathological processes may interact in individuals with T2DM.

Overall, these findings point toward a framework in which vascular and neurodegenerative pathways overlap rather than operate independently.

### Mechanistic integration: from metabolism to neurodegeneration

Insulin resistance may play a key role in linking T2DM to neurodegeneration. Disruptions in insulin signaling can impair neuronal glucose utilization, synaptic plasticity, and mitochondrial function [15, 19]. In addition, hyperinsulinemia may interfere with amyloid-beta clearance through competition for insulin-degrading enzyme, while altered intracellular signaling pathways may contribute to tau phosphorylation. These mechanisms provide a possible biological link between metabolic dysfunction and core AD pathology.

Chronic hyperglycemia is also associated with systemic inflammation, oxidative stress, and accumulation of advanced glycation end-products [17]. These processes can increase blood–brain barrier permeability and promote microglial activation, which may accelerate neuronal injury. Biomarker studies have reported elevated inflammatory mediators in individuals with T2DM who also show signs of cognitive impairment [28].

Furthermore, diabetes-related endothelial dysfunction and microvascular remodeling have been linked to white matter hyperintensities and reduced cerebral perfusion [18]. Experimental studies also suggest that hyperamylinemia may contribute to microvascular brain injury and white matter disease [16]. These mechanisms closely align with the stronger epidemiologic association observed for vascular dementia.

Together, these pathways likely interact rather than acting independently. Insulin resistance, inflammation, oxidative stress, and vascular injury may reinforce each other over time, which mirrors the epidemiologic observation that earlier onset, longer duration, and metabolic instability are associated with greater risk.

### Pharmacologic implications

Recent pharmacoepidemiologic studies suggest that dementia risk may vary across different glucose-lowering medication classes. For example, initiation of SGLT2 inhibitors has been associated with lower dementia risk compared with DPP-4 inhibitors in a population-based cohort [20]. Additionally, meta-analyses indicate that SGLT2 inhibitors and GLP-1 receptor agonists may be linked with reduced risk of dementia or cognitive decline [21, 22].

Although these findings are based on observational data, they align with proposed mechanisms related to improved metabolic signaling, vascular protection, and reduced inflammation. However, randomized trials with cognitive outcomes are needed to determine whether these associations reflect causal effects. These observational comparisons are subject to confounding by indication and channeling bias, as medication selection may reflect underlying cardiovascular or metabolic risk profiles.

### Methodological considerations

There is considerable variability across studies in terms of how T2DM exposure, disease duration, and dementia outcomes are defined. Differences in analytic methods and follow-up periods also make direct comparisons challenging. In addition, factors such as competing mortality, reverse causation, and residual confounding by factors such as educational attainment, socioeconomic status, obesity, and physical activity level, each an established risk factor for both T2DM and dementia, which were inconsistently adjusted for across the included studies. Because individuals with T2DM have elevated cardiovascular mortality, competing risk of death may lead to underestimation or distortion of observed dementia incidence in some cohorts.

Despite these limitations, the overall direction of association is consistent across diverse populations and study designs. Many recent cohorts have also incorporated competing risk models and cumulative exposure measures, which strengthen the interpretation of life-course risk patterns.

### Strengths and limitations of the evidence base

A key strength of this review is the integration of duration-specific epidemiologic gradients with mechanistic and pharmacoepidemiologic data within a life-course conceptual model. In this context, the evidence linking midlife T2DM to dementia is strengthened by consistent findings across diverse populations, long follow-up periods, and graded exposure–response relationships. However, most data derive from observational studies and remain vulnerable to residual confounding, reverse causation, and survival bias. Dementia subtype classification varies substantially across cohorts, and administrative coding may underestimate mixed pathology. Furthermore, confounding by indication may influence pharmacoepidemiologic findings. These limitations should temper causal interpretation.

In addition, the proportion of participants treated with insulin was inconsistently reported across the included studies, and most analyses did not stratify dementia risk by insulin use. Because insulin therapy is both a marker of more advanced disease and a principal driver of severe hypoglycemia, this limits our ability to separate the contribution of cumulative glycemic burden from that of treatment intensity. This distinction is likely important: in a nationwide Korean cohort, insulin-treated T2DM was associated with approximately twice the dementia risk of T2DM managed with oral agents (HR 2.14 vs. 1.29), a risk comparable to that observed in type 1 diabetes [6].

### Clinical and public health implications

The convergence of epidemiologic and mechanistic evidence suggests that preventing and managing T2DM during midlife could play an important role in reducing dementia risk. The 2024 Lancet Commission identifies diabetes as a key modifiable risk factor within a life-course approach to dementia prevention [1].

In clinical practice, management strategies must balance reducing hyperglycemia with avoiding hypoglycemia, particularly in older adults [14]. Individualized glycemic targets, careful medication selection, and comprehensive management of vascular risk factors may all contribute to lowering cognitive risk.

From a public health perspective, delaying the onset of T2DM or reducing cumulative glycemic burden during midlife could have meaningful effects on future dementia incidence.

## Conclusion

Midlife type 2 diabetes is consistently associated with an increased risk of late-life dementia. This risk is not uniform and appears to increase with earlier age at onset, longer duration of disease, greater cumulative glycemic burden, and the occurrence of severe hypoglycemia. Together, these patterns support a life-course model in which prolonged metabolic and vascular dysregulation is associated with neurodegenerative and cerebrovascular changes.

Mechanistic evidence involving insulin resistance, inflammation, oxidative stress, and microvascular injury provides biological plausibility for these epidemiologic findings. Emerging pharmacoepidemiologic data also suggest that certain glucose-lowering therapies may influence cognitive risk, although further research is needed to confirm these effects. Because most available evidence is observational, causal inference remains limited, and randomized trials with cognitive outcomes are needed.

These findings have several practical implications for clinical care and early intervention. First, because risk appears to accumulate from the time of onset, early detection of dysglycemia is a logical starting point: the U.S. Preventive Services Task Force recommends screening for prediabetes and T2DM in adults aged 35 to 70 years with overweight or obesity [29], and structured lifestyle intervention in adults with prediabetes reduced progression to T2DM by 58% in the Diabetes Prevention Program [30]. Second, individuals diagnosed with T2DM in midlife, particularly before age 60, may warrant earlier and more sustained management of glycemic and vascular risk factors given their longer projected exposure [26, 27]. Third, routine screening for cognitive impairment with brief validated instruments (e.g., Mini-Cog or the Montreal Cognitive Assessment) is recommended for adults with diabetes aged 65 years and older [14]; detection of impairment should prompt simplification of glucose-lowering regimens and closer monitoring for hypoglycemia, given its bidirectional relationship with cognitive decline. Fourth, among individuals treated with insulin or sulfonylureas, systematic review of hypoglycemia history and, where available, continuous glucose monitoring may help reduce hypoglycemic exposure and glycemic variability. Finally, glycemic history itself (age at diagnosis, disease duration, longitudinal HbA1c trajectories, and prior severe hypoglycemia) could be incorporated into dementia risk stratification to identify those most likely to benefit from early preventive intervention.

Overall, the evidence highlights midlife as a critical window for intervention. Preventing T2DM onset, maintaining stable metabolic control, minimizing hypoglycemia, and addressing vascular risk factors may collectively help reduce the future burden of dementia. 

## Data Availability

No datasets were generated or analysed during the current study.
